# Increased Water Storage in the Qaidam Basin, the North Tibet Plateau from GRACE Gravity Data

**DOI:** 10.1371/journal.pone.0141442

**Published:** 2015-10-27

**Authors:** Jiu Jimmy Jiao, Xiaotao Zhang, Yi Liu, Xingxing Kuang

**Affiliations:** Department of Earth Sciences, The University of Hong Kong, Pokfulam Road, Hong Kong, China; Institute of Tibetan Plateau Research, CHINA

## Abstract

Groundwater plays a key role in maintaining the ecology and environment in the hyperarid Qaidam Basin (QB). Indirect evidence and data from sparse observation wells suggest that groundwater in the QB is increasing but there has been no regional assessment of the groundwater conditions in the entire basin because of its remoteness and the severity of the arid environment. Here we report changes in the spatial and temporal distribution of terrestrial water storage (TWS) in the northern Tibetan Plateau (NTP) using Gravity Recovery and Climate Experiment (GRACE) data. Our study confirms long-term (2003–2012) TWS increases in the NTP. Between 2003 and 2012 the TWS increased by 88.4 and 20.6 km^3^ in the NTP and the QB, respectively, which is 225% and 52% of the capacity of the Three Gorges Reservoir, respectively. Soil and water changes from the Global Land Data Assimilation System (GLDAS) were also used to identify groundwater storage in the TWS and to demonstrate a long-term increase in groundwater storage in the QB. We demonstrate that increases in groundwater, not lake water, are dominant in the QB, as observed by groundwater levels. Our study suggests that the TWS increase was likely caused by a regional increase in precipitation and a decrease in evaporation. Degradation of the permafrost increases the thickness of the active layers providing increased storage for infiltrated precipitation and snow and ice melt water, which may also contribute to the increased TWS. The huge increase of water storage in the NTP will have profound effects, not only on local ecology and environment, but also on global water storage and sea level changes.

## Introduction

The Tibetan Plateau (TP), as the Third Pole of the world [1], is characterized by 12,000 km^3^ of glaciers and 122.2×10^4^ km^2^ of permafrost [2]. Melt water from this region ensures permanent flow of Asia's major river systems, so the TP is called the water tower of Asia [3]. Global climate changes cause glacier retreat, snow melt and permafrost degradation [4–6], which then influence water storage distribution in the TP. Eventually these changes affect the livelihood of over 1.3 billion people and various ecosystems fed by river water that originates in the TP.

Water storage in the TP has been studied using the Gravity Recovery and Climate Experiment (GRACE) data ever since the data became available in August 2002. Conclusions regarding changes in the water storage in the TP vary, depending on the region studied and the periods over which the studies were carried out. The first analysis of water storage in the TP using GRACE data, which was based on the time period from 2002 to 2006, concluded that the TP had an area-average reduction in water thickness of 0.031±0.019 cm/month [7]. Another study based on the GRACE data between 2002 and 2008 for areas in India close to the southwestern edge of the TP demonstrated huge groundwater depletion in that area that may be due to over consumption of groundwater for irrigation and other anthropogenic uses [8]. Similar conclusions were also reached by other studies using GRACE data [9,10]. For example, a study in the source region of the Yellow River over the period 2003–2008 concluded that water storage in this region increased by 0.51 mm/month, probably as a result of permafrost degradation and the resulting increase in the thickness of the active layer [11]. Using GRACE data, Moiwo et al. [12] analyzed changes in the total water storage over the entire TP from 2003 to 2008 and concluded that there was a water storage loss. Matsuo and Heki [13] investigated the negative gravity trend in the region and concluded that it is due to substantial melting of glaciers. They discussed three factors that may cause uncertainty in their estimates: groundwater decline in northern India, tectonic uplift and glacial isostatic adjustment. They estimated that the average rate of ice loss in the high mountains of Asia is 47±12 km^3^/y, equivalent to ~0.13±0.04 mm/y sea level rise. In a study of the contributions of melting glaciers and ice caps in the high mountains of Asia on global sea level rise, Jacob et al. [14] concluded that the water mass is increasing in Tibet and Qilian Shan, even though there is an overall decrease over the entire region. Jacob et al. [14] attributed this mass increase to an increase in glacier mass, whereas Zhang et al. [15] concluded that the increase is caused by water accumulation in lakes.

Most studies of water storage in the TP have focused on glaciers, snow packs and lakes and rivers, where the impact of groundwater storage has been generally ignored. Recent studies in Nepal have demonstrated that the volume of water flowing through basement aquifers can be approximately six times higher than the contribution of melt water from glaciers and snow melt to river discharge [16], suggesting that groundwater is an extremely important component of the hydrology of the TP.

**Fig 1:**
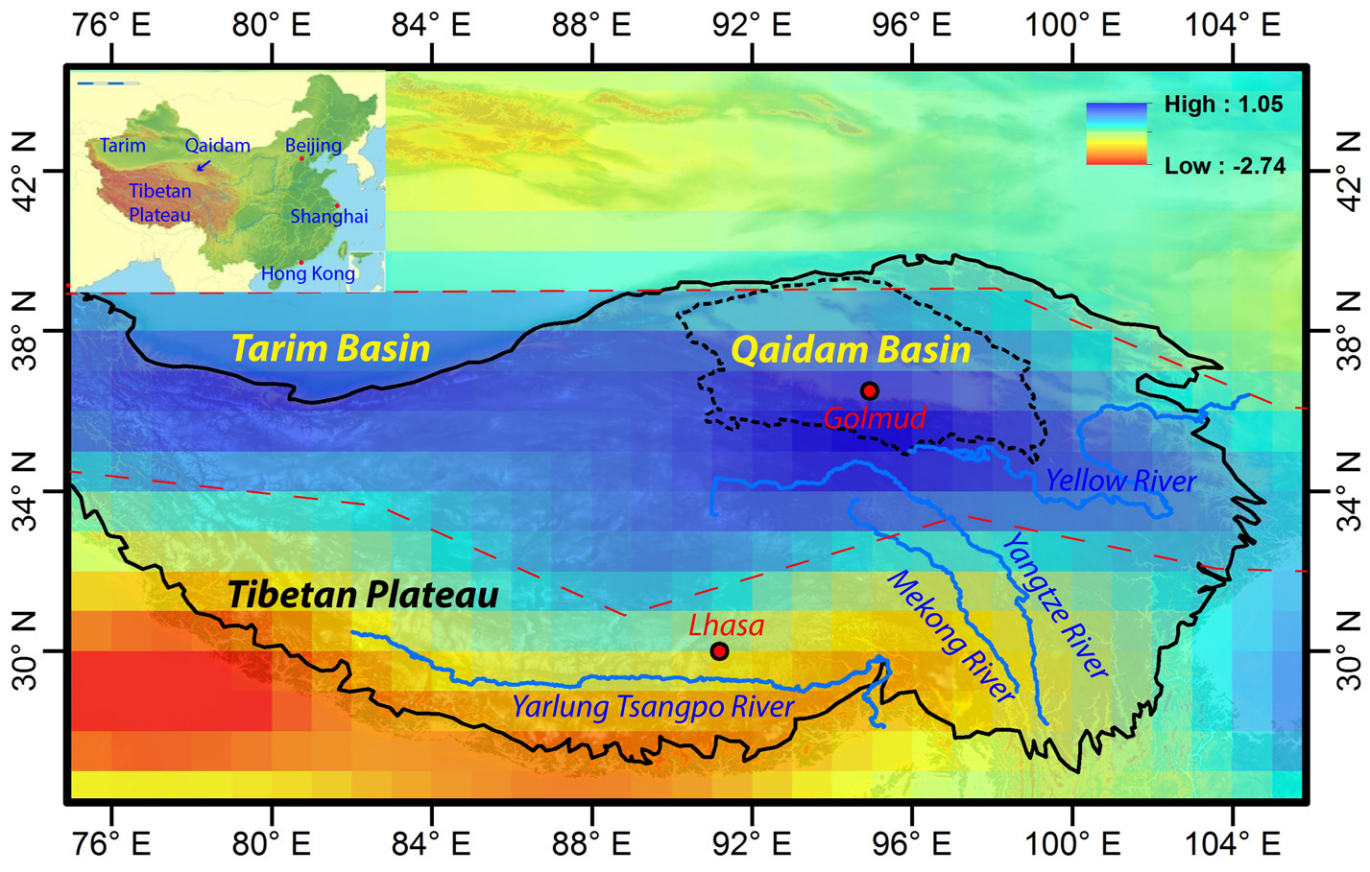
Spatial distribution of changing rates of TWS (mm/month) in the TP from 2003 to 2012, indicating an increase in water storage in the NTP, especially in the QB. The background shows the topography of the area.

**Fig 2:**
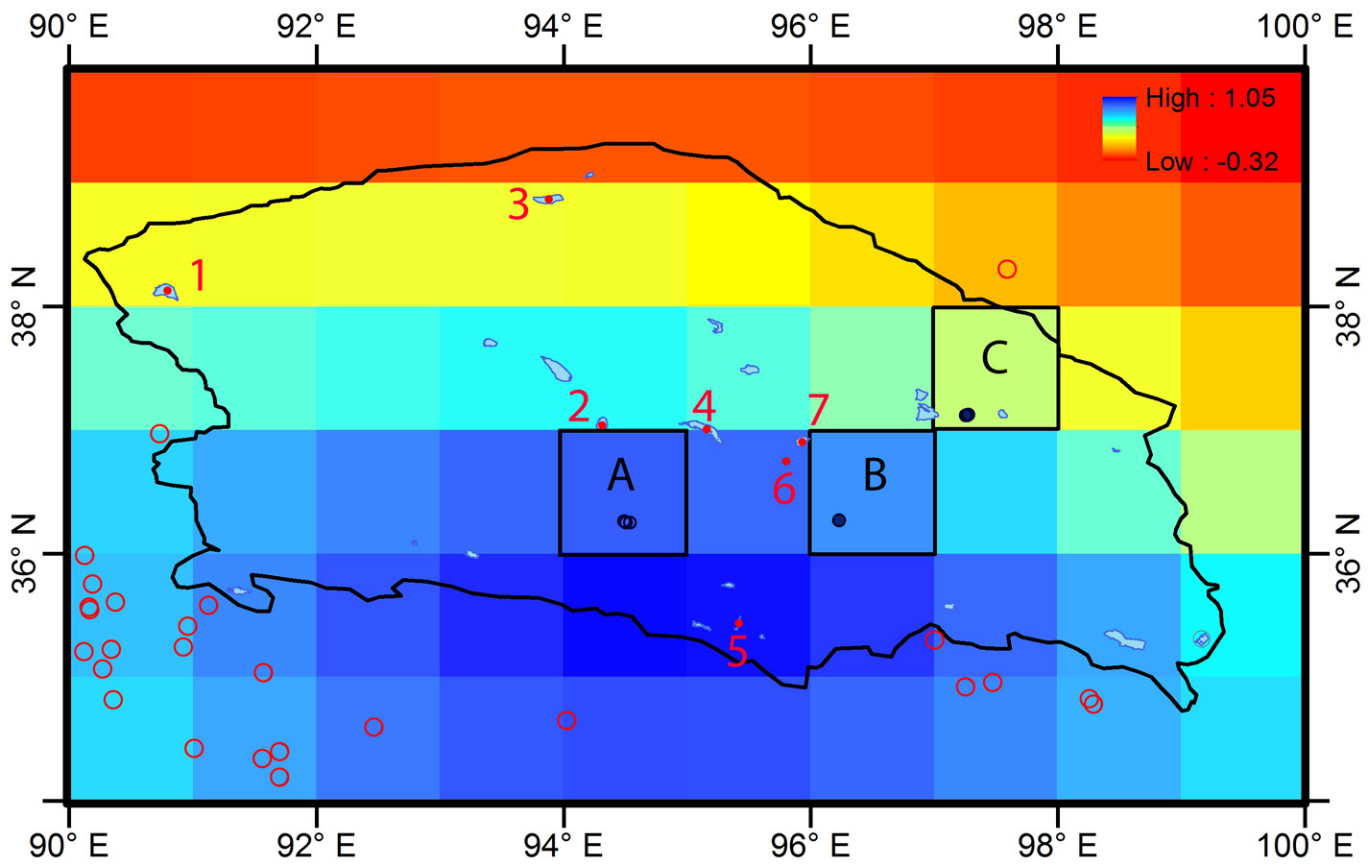
Changes in TWS from 2003 to 2012 in the QB and the locations of lakes. Lakes inside the QB with water level data are numbered as shown in Table 1 and those around the QB are marked in red circles. Three grids with observation wells (represented by black circles) are marked as A, B, and C.

Although many studies have focused on different parts of the TP, no studies have been undertaken on water storage in the QB, a huge arid and semi-arid basin on the northern edge of the TP (Fig 1). The basin extends about 800 km in an E-W direction, is about 350 km wide and has a total area of approximately 256,000 km^2^. It has an elevation of 2600–3000 m [17], and is characterized by an annual potential evaporation up to 3,700 mm [17] and an overall annual precipitation of less than 300 mm [18]. The average annual temperature ranges from 1.2°C to 4.3°C. It is a closed basin surrounded on all sides by mountains; the rivers that originate in the mountains discharge into salt lakes and saline swamps within the basin. Rivers and lakes mostly occur in the eastern part of the basin, and the western part has very little surface water. There are 9 major permanent rivers in the basin, each with a discharge greater than 1 × 10^8^ m^3^/y [17]. Most of the rivers originate on the southern mountain slopes and radiate into the basin; the longest river extends for about 435 km [17]. The river water is derived mostly from glaciers and snow packs. Most of the river water eventually becomes groundwater downstream. There are 25 lakes in the QB, with areas ranging from 1.5 to 135 km^2^ [17] (Fig 2). Most of them are salt lakes due to long-term evaporation. About a decade ago, there were major concerns about environmental and ecological degradation due to land desertification and salinization caused by the dry climate, deforestation, overgrazing, as well as unsustainable use of both groundwater and surface water for irrigation and industrial purposes [19–21]. However, the situation has improved significantly in recent years [22].

The aim of this study is to examine the distribution of changes in the TWS in the TP, with a focus on the QB. Change in groundwater storage in the QB is estimated from the TWS. This change is then compared with the water level data from limited long-term observation wells in the QB. The mechanisms responsible for the changes in groundwater storage are examined, and the implications of these changes on water resources and the environment are discussed.

## Materials and Methods

The Gravity Recovery and Climate Experiment (GRACE) mission, launched by NASA and the German Aerospace Centre in May 2002, features twin co-orbiting satellites that in tandem measure the Earth’s gravity field with unprecedented accuracy and can provide vertically integrated TWS change after adjusting for nonhydrologic effects [12,23]. Data representing a total of 115 monthly TWS anomalies from January 2003 to December 2012 were used in this study [24,25]. The TWS values estimated from GRACE are presented as TWS anomalies, which are calculated as the original monthly values minus their mean over the period from 2004 to 2009 (http://grace.jpl.nasa.gov/data/gracemonthlymassgridsland/). To be consistent with the TWS data, all other data series, such as soil water and groundwater, are calculated as anomalies over their average values for the same period.

Monthly data from the Global Land Data Assimilation System (GLDAS) for the period January 2003 to December 2012, the same period as the with GRACE data, were also used in this study. The GLDAS data, which are presented on global 1°×1° grids, simulate soil-water fields and integrate the effects of precipitation, solar radiation, air temperature and other meteorological factors [8].

The total area of the 25 lakes in the QB is 1612.6 km^2^, as estimated from Landsat satellite images in 2004. No gauges are available to monitor the lake water levels. Lakes with ICESat data from 2003 to 2009 in the TP, including these seven in the QB, were studied by Phan et al. [26] to estimate the water level. Water-level changes from 2003 to 2009 in these seven lakes were taken from Phan et al. [26] and used here to estimate changes in their water inventory (Table 1). The changing rate of the inventory of total lake area is estimated approximately from the changing rate of inventory in the seven lakes, details of which are explained later.

**Table 1:** Locations of lakes and changes in their water levels and inventory (NA: lake names are not available).

No.	Lat.	Lon.	Name	Area (km^2^)	Elevation change (m/y)	Inventory change (km^3^/y)
1	38.1146	90.7815	Gasi Kule	124.335	0.002	0.000249
2	37.0435	94.3903	NA	13.55	-0.008	-0.00011
3	38.8685	93.8782	Suqian	102.235	-0.022	-0.00225
4	36.9781	95.2047	Dabuxun	290.922	0.034	0.009891
5	35.439	95.4182	NA	7.991	0.005	4E-05
6	36.7276	95.8222	Nanhuoluxun Tso	4.108	0.003	1.23E-05
7	36.9035	95.9502	NA	92.946	0.012	0.001115

In the QB, there are three grids A, B and C with long-term groundwater monitoring wells (Fig 2). Grids A, B, and C have 3, 1, and 3 observation wells, respectively. The water levels in these grids have been compared with the groundwater storage variations in the grids. The groundwater levels were converted into anomaly values in the same manner used to calculate the TWS anomalies.

## Results

### 3.1 Spatial Distribution of TWS

TWS changes in the TP, including the QB, from 2002–2012 were examined. Near the southern boundary of the TP, there is an obvious decrease in TWS (Fig 1). The decrease in the bottom left corner is believed due to groundwater depletion [8]. The decrease along the Yarlung Tsangpo river was caused by melting of snow and glacial ice, with the resulting water being drained away by the river. The TWS in a large part of the TP, especially in the NTP, however, is increasing. Most of the grids with storage increases are located between latitude 34° N and 38° N. The grids in the southern part of the QB have the greatest increase in storage.

Fig 2 shows the detailed TWS variations in the QB. The total TWS increase in the QB (inside the areas bounded by the black line) was calculated. When a grid is located at the boundary, only the TWS change in the portion of the grid within the boundary was included, and was calculated as the product of the TWS of the grid and the ratio of the area inside of the boundary to the total area of the grid. Most of the grids with TWS increase are located in the NTP and the total TWS increase in the areas with TWS increase (see the area bounded by the red broken lines in Fig 1) is 88.39 km^3^, which is 2.25 times the capacity of Three Gorges Reservoir with a water capacity of 39.3 km^3^ when the water level reaches 175 m [27]. Fig 2 shows the detailed distribution of the grids with TWS increase around the QB; the grids around the south boundary of the QB have the greatest increase in the TWS (Figs 1 and 2). The total TWS increase in the QB is 20.57 km^3^ (Table 2), which is 0.52 times the capacity of Three Gorges Reservoir.

**Table 2:** Variations of water components of TWS and precipitation around the QB between 2003 and 2012. The brackets in the second column show the percentage contribution for each component of the TWS. Errors are calculated except for the data related to lakes because the lake level from [26] had no error information.

Water component	Total variation (km^3^)	Annual changing rate (km^3^/y)
TWS	20.57±2.82	2.24±0.28
Soil moisture	3.09±0.98 (15%)	0.53±0.1
Snow water equivalent	0.68±0.66 (3.3%)	0.06±0.07
Lakes	0.23 (1.1%)	0.02
GW	16.57±1.36 (80.6%)	1.62±0.14
Rainfall	20.35±7.16	2.03±0.72

### 3.2 Temporal Changes of TWS and Its Components

The TWS in the TP generally consists of storage in snow, glaciers, rivers, lakes, soil water and groundwater [8,28,29] and it is important to know which of these components dominate the changes in the TWS. The primary aim of this study was to understand the temporal and spatial distribution of groundwater storage in the QB. If other components are known, groundwater storage can be estimated by deducting them from the TWS.

The contributions from glaciers, rivers and lakes are difficult to estimate. In a similar study about TWS in Punjab in India southwest of the Himalayas [8], the glacial component was ignored because its contribution to the TWS was estimated to be very small. In the QB, we also ignored the glacial component of the TWS. Even in the source regions of the Yellow and Yangtze Rivers, which are much farther south than the QB and have higher elevations, the glacier areas are only 0.11% and 0.95% of the total source areas, and the contributions of glacial melt water to the rivers are only 0.8 and 6.5% respectively [30]. The QB has many fewer glaciers than the source regions of these two rivers and a previous study concluded that the maximum glacial contribution to the rivers in the QB is < 0.1% of the river discharge [31].

In most cases, groundwater is the major component of the TWS, even in a major river catchment, because the area with aquifers is always much larger than the river channels with surface water. In the mountainous areas of a river catchment, groundwater is even more important component of TWS variations because the water table there is deep and the large subsurface storage capacity holds the infiltrated water longer before releasing it to streams [32]. A study on the role of groundwater in the Amazon Basin water cycle concluded that contributions of subsurface water accounted for 95% of the observed variations in TWS and that only 5% was due to surface water in river channels [32]. The rivers in the QB are sparse and very small, so water storage variations in these river channels are ignored in the following discussion.

If contributions from glaciers and rivers are ignored, the TWS variability is assumed to be mainly caused by the variations of groundwater (*GW*), soil moisture (*SM*) from 0 to 2.5 m below ground surface, snow water equivalent (*SWE*) [8,28,29], and lake water (*LW*). Thus, groundwater storage changes are estimated as:

GW=TWS−(SM+SWE+LW) (1)

The soil moisture (*SM*) and snow water equivalent data (*SWE*) can be obtained from the GLDAS data.

Water-level changes in lakes of the QB, which range from -0.022 to 0.034 m/y, are very small compared to many lakes in the inner TP [26,33]. Water levels are increasing in five of the seven lakes included in this study, and decreasing in two. The total area of the seven lakes is 636.1 km^2^ and the changing rate of the total inventory these lakes is 0.00895 km^3^/y. According to this area and changing ratio, the changing rate for the total inventory in the lake area of 1612.5 km^2^ in the whole QB is estimated to be 0.0227 km^3^/y. The spatial distribution of the seven lakes is shown in Fig 2. The average rate of change in water levels for the period 2004–2008 estimated by Phan et al. [26] from the ICESat data is used to approximate the inventory changes in all 25 lakes from 2003 to 2012, which we have calculated to be 0.023 km^3^/y (Table 2).

**Fig 3:**
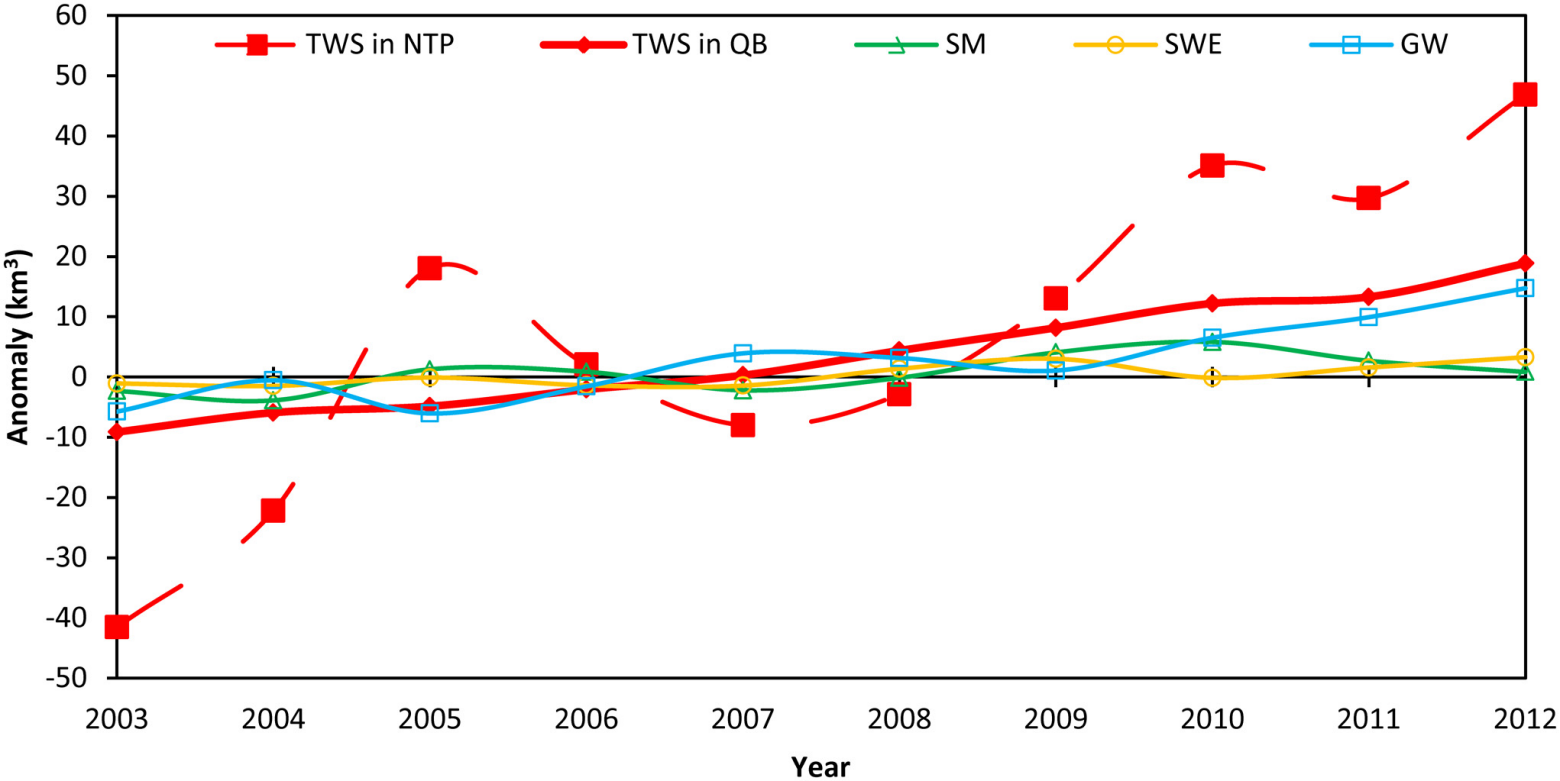
Changes in the TWS in the NTP and QB, and contributions to the TWS from its components (soil moisture, snow water equivalent and groundwater) in the QB from 2003 to 2012.

The temporal changes of the TWS in the NTP and the QB are compared in Fig 3, which shows that the TWS in both is increasing with time. In the NTP, changes in TWS fluctuate considerably, whereas those in the QB shows a very steady increase. Fig 3 also shows the temporal changes of the components contributing to the TWS in the QB from 2002 to 2012. Variation of lake water is very small and are not included in the figure. As the TWS increases steadily, groundwater and soil water moisture also increase, although with some fluctuations.

Using the linear trends of TWS in the QB and information on its various components presented in Fig 3, we calculated the water storage changes from 2003 to 2012 (Table 2). In the past 10 years or so, the TWS and groundwater storage increased by 20.57 km^3^ and 16.57 km^3^, respectively, at a rate of 2.24 km^3^/y and 1.62 km^3^/y, respectively. Groundwater storage accounts for 80.6% of the TWS, whereas lake water increased by 0.23 km^3^ and only accounted for about 1.1% of the TWS. This suggests that groundwater is the major component in the TWS in the QB. This conclusion differs from that of a study on the relationship between TWS and lake storage in the TP [15]. Those workers concluded that increases in TWS were almost totally due to increases in lake levels as a result of increased precipitation [15].

## Discussion

### 4.1 Comparison of Estimated Changes in Groundwater Storage and Observed Groundwater Levels

Groundwater increases in the QB has been reported in the recent years. As stated in an official report [34], groundwater levels started to rise in the southern part of the QB in 2009 as a result of continuously increasing precipitation and river runoff. An increase of 3.07 m in the groundwater level was reported for the period January to November, 2009 [22], although no information was provided on the exact location and the size of the area where this increase occurred. Flooding was reported due to high groundwater levels, which created problems for the local residents. The overall effect of the groundwater increase, however, was positive because more water was available for consumption and a significant expansion of wetlands reduced erosion of valuable soil [22].

**Fig 4:**
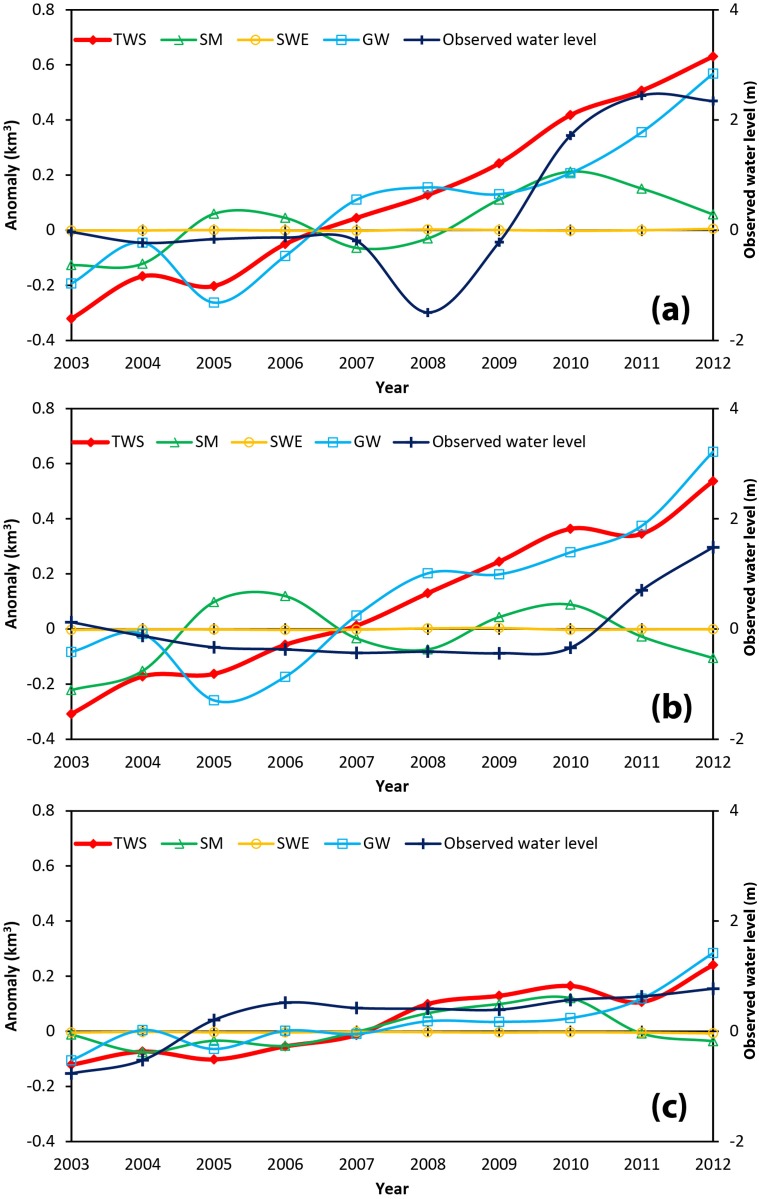
Changes of the TWS, its components, and observed groundwater levels in grids A (a), B (b), and C (c).

The temporal changes of the groundwater levels as well as the TWS and its components, are presented in Fig 4. Note that in these grids, there are no major lakes or rivers so surface water is ignored. As can be seen from Fig 4, the TWS and its components fluctuate more significantly than the corresponding values for the QB in Fig 3. This is because the values in Fig 3 are averages from the entire QB, whereas those in Fig 4 represent only the values in individual grids. Changes in TWS and groundwater storage in grids A and B are both significant but those in grid C are much smaller. Groundwater is the dominant component of the TWS in all three grids. The overall increasing trend of groundwater storage is similar to that of the observed water levels, suggesting that the groundwater storage increase estimated from the TWS is reasonable. However, the details of the trends in water level and groundwater storage are not directly comparable. This is because the observed water level represents a well or the average water level from a few wells in a grid, which are controlled by local conditions such as the geology and extent of groundwater usage, whereas the groundwater storage variations represent average groundwater changes over an entire grid.

### 4.2 Factors Causing the Temporal Changes in Groundwater Storage and TWS

#### 4.2.1 TWS and precipitation

It is natural to assume that the observed increases in TWS may be related to increased melting of snow and glacial ice due to rising temperatures [14,35]. The water produced by this process moves from high to low elevations, leading to local redistribution of water storage. When melt waters flow into lakes, the water storage is locally increased, whereas when the melt waters flow into rivers they are carried downstream, leading to redistribution of water storage over relatively large areas. However, most of the rivers in the QB are relatively short considering the large scale of the study (1 grid≈100×100 km^2^) involved in the TWS calculations, thus the redistribution of melt water storage caused via rivers may not be as significant as expected. If melting of snow and glacial ice causes an increase TWS in downstream areas, there must be a decrease in the areas covered by ice and snow. Thus, a regional increase in TWS over the entire QB, must be due to other factors.

**Fig 5:**
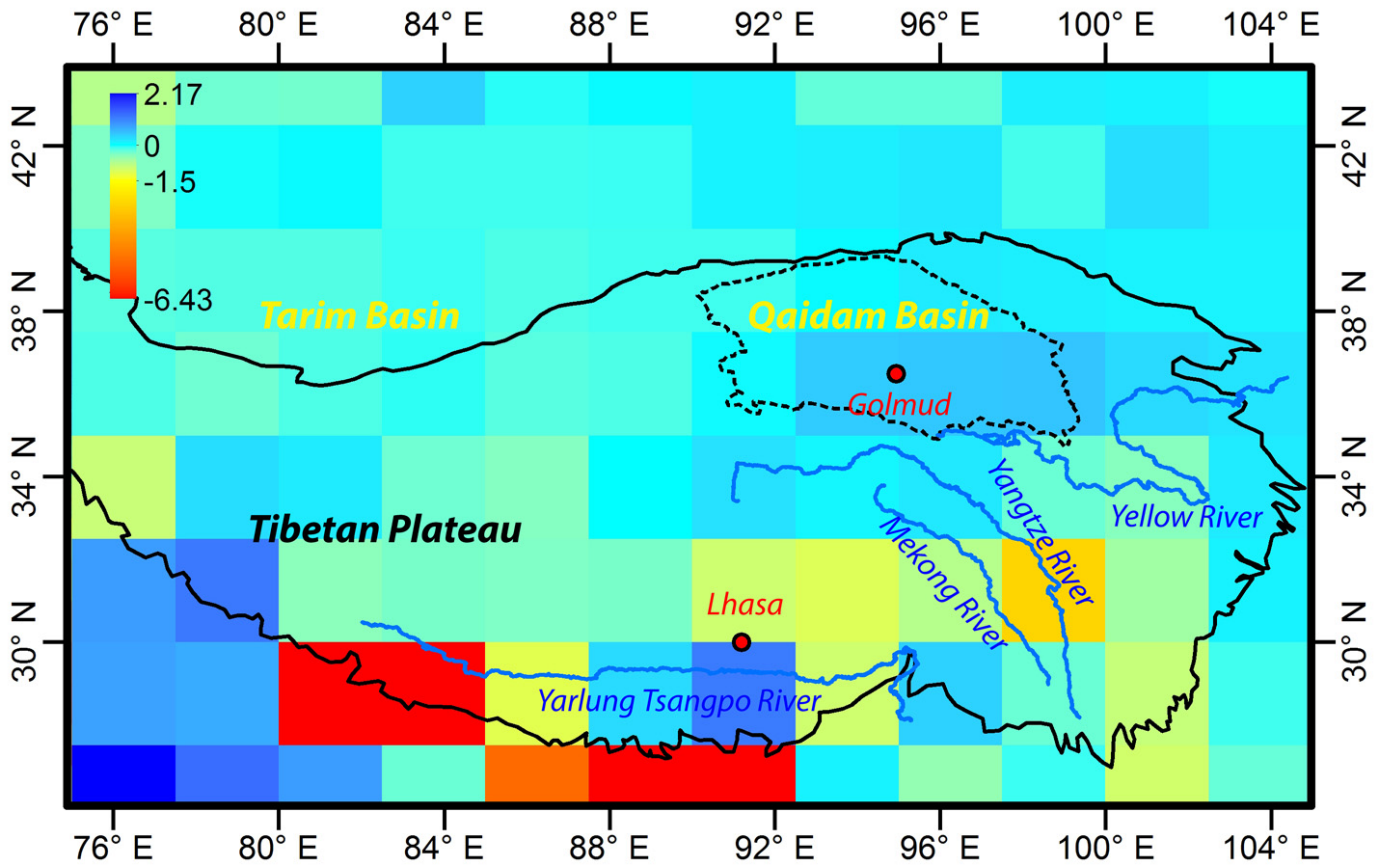
Spatial distribution of changes in precipitation (mm/month) in the TP and surrounding regions from 2003 to 2012 calculated by GPCP.

Changes in the TWS must be closely related to factors such as precipitation and evaporation. Fig 5 shows changes in precipitation over the region (mm/y) from 2003 to 2012 calculated by GPCP (Global Precipitation Climatology Project). While overall precipitation south of latitude 35°N is decreasing and the pattern of change is complicated, precipitation north of 35° N is clearly increasing and the rate of increase is greatest in the grids near the southern boundary of the QB. This pattern was also confirmed by gauge-based precipitation [36]. There is a strong positive correlation between precipitation distribution and TWS in the QB, suggesting that variations in precipitation are a driving factor in TWS. The total precipitation increase in the QB from 2003 to 2012 is estimated to be 20.35 km^3^ (Table 2), which is nearly identical with the TWS increase of 20.57 km^3^.

Evaporation is another factor that may influence the TWS. Overall the potential evaporation increased in most of the TP between 1996 and 2010 due to an increase in temperature but it decreased slightly in the QB and surrounding areas [36,37], probably because both the days of sunlight and the wind speed decreased [38,39,40]. Thus, we attribute the increase in TWS in the QB to both an increase in precipitation and a decrease in evaporation.

#### 4.2.2 TWS and permafrost

A warming climate not only accelerates melting of glaciers and perennial snow cover, but also triggers permafrost degradation. The depth of the permafrost active layer in the TP, including the QB, has been increasing during the past few decades [6,41]. Only seasonal frozen ground is present in the central QB but permafrost persists near the southern and northern mountain ranges, especially in the area southeast of Golmud [41,42], which is also the area with the largest increase in TWS.

Typically, degradation of permafrost should lead to a decrease in groundwater storage in the shallow soil, which then becomes drier due to increases in the thickness of the unsaturated zone and/or reduction in the thickness of the shallow unconfined aquifer. However, an increase in the thickness of the unsaturated zones provides more room for infiltrating precipitation, leading to an increase in water storage in the shallow unconfined aquifers [43,44,45]. This may be also a factor related to the increased groundwater storage and TWS in the region.

Typically permafrost does not exist below lakes and deeply incised river channels [46], and there is an active hydraulic connection between river and lake water and the unfrozen soil, or talik, below the river or lake bottom. Permafrost degradation releases originally frozen groundwater to flow to the lakes or rivers, but at the same time, the increased talik areas may also lead to a loss of surface water to the shallow unconfined aquifer or a deep confined aquifer below the lakes or rivers because the surface water bodies are usually connected with deep aquifers through the talik zones. Consequently, an increase in precipitation eventually leads to an increase in water storage in both shallow and deep aquifers.

## Summary and Conclusions

This study demonstrates that there is a significant increase in the TWS in the NTP, especially in the QB and its surrounding areas. Between 2003 and 2012, the TWS increase in the NTP (including its neighboring area) and the QB amounted to 88.4 and 20.6 km^3^, respectively, which is 225% and 52% of the capacity of the Three Gorges Reservoir. As water trapped in the endorheic basins in Australia can lead to global sea level reduction [47], such a huge increase in water storage in the NTP will have profound impacts both locally and globally.

In the QB, groundwater storage accounts for 80.6% of the increase in TWS. This increase in groundwater storage has been confirmed by measurements in monitoring wells in the region. The observed increase in water storage is mainly driven by a significant increase in precipitation and a slight decrease in evaporation. An increase in thickness of the active layer due to permafrost degradation also provides room for infiltration of increased precipitation to the shallow and deep aquifers, leading to the observed increase in groundwater storage. This increase in groundwater storage will play an important role in maintaining and improving the ecology and environment in the hyperarid Qaidam Basin.

## References

[1] Qiu J (2008) The third pole. Nature 454: 393–396. 10.1038/454393a 18650887

[2] Guo DL, Wang HJ, Li D (2012) A projection of permafrost degradation on the Tibetan Plateau during the 21st century. Journal of Geophysical Research-Atmospheres 117: D05106.

[3] Xu XD, Lu CG, Shi XH, Gao ST (2008) World water tower: An atmospheric perspective. Geophysical Research Letters 35: L20815.

[4] Guo DL, Wang HJ (2013) Simulation of permafrost and seasonally frozen ground conditions on the Tibetan Plateau, 1981–2010. Journal of Geophysical Research-Atmospheres 118: 5216–5230.

[5] Wei YQ, Fang YP (2013) Spatio-temporal characteristics of global warming in the Tibetan Plateau during the last 50 years based on a generalised temperature zone—elevation model. PLoS ONE 8: e60044 10.1371/journal.pone.0060044 23565182 PMC3615011

[6] Li R, Zhao L, Ding YJ, Wu TH, Xiao Y, Du EJ, et al (2012) Temporal and spatial variations of the active layer along the Qinghai-Tibet Highway in a permafrost region. Chinese Science Bulletin 57: 4609–4616.

[7] Muskett RR (2008) GRACE equivalent water mass balance of the Himalayas and Tibet Plateau region. EGU General Assembly 2008.

[8] Rodell M, Velicogna I, Famiglietti JS (2009) Satellite-based estimates of groundwater depletion in India. Nature 460: 999–1002. 10.1038/nature08238 19675570

[9] Tiwari VM, Wahr J, Swenson S (2009) Dwindling groundwater resources in northern India, from satellite gravity observations. Geophysical Research Letters 36: L18401.

[10] Fu YN, Freymueller JT (2012) Seasonal and long-term vertical deformation in the Nepal Himalaya constrained by GPS and GRACE measurements. Journal of Geophysical Research-Solid Earth 117: B03407.

[11] Xu M, Ye BS, Zhao QD, Zhang SQ, Wang J (2013) Estimation of water balance in the source region of the Yellow River based on GRACE satellite data. Journal of Arid Land 5: 384–395.

[12] Moiwo JP, Yang YH, Tao FL, Lu WX, Han SM (2011) Water storage change in the Himalayas from the Gravity Recovery and Climate Experiment (GRACE) and an empirical climate model. Water Resources Research 47: W07521.

[13] Matsuo K, Heki K (2010) Time-variable ice loss in Asian high mountains from satellite gravimetry. Earth and Planetary Science Letters 290: 30–36.

[14] Jacob T, Wahr J, Pfeffer WT, Swenson S (2012) Recent contributions of glaciers and ice caps to sea level rise. Nature 482: 514–518. 10.1038/nature10847 22318519

[15] Zhang G, Yao T, Xie H, Kang S, Lei Y (2013) Increased mass over the Tibetan Plateau: From lakes or glaciers? Geophysical Research Letters 40: 2125–2130.

[16] Andermann C, Longuevergne L, Bonnet S, Crave A, Davy P, Gloaguen R (2012) Impact of transient groundwater storage on the discharge of Himalayan rivers. Nature Geoscience 5: 127–132.

[17] China Geological Survey (2008) Investigation and Assessment of Groundwater Resources and Their Environment Issues in the Qaidam Basin (in Chinese with extended English Summary). Beijing: Geological Publisher.

[18] Wang X, Yang M, Liang X, Pang G, Wan G, Chen X, et al (2014) The dramatic climate warming in the Qaidam Basin, northeastern Tibetan Plateau, during 1961–2010. International Journal of Climatology 34: 1524–1537.

[19] Qian KZ, Wan L, Wang XS, Lv JJ, Liang SH (2012) Periodical characteristics of baseflow in the source region of the Yangtze River. Journal of Arid Land 4: 113–122.

[20] Dong ZB, Wang XM, Liu LY (2000) Wind erosion in arid and semiarid China: an overview. Journal of Soil and Water Conservation 55: 439–444.

[21] Bao Z, Dong X, Guo S, Xu W (2010) Main problem and solutions of Chaidamu basin groundwater resources exploitation. Joumal of Qinghai University (Nature Science) (in Chinese with English abstract) 28: 54–59.

[22] Jiang CR (2009) China's Qaidam Basin sensitive to global warming People's Daily Online. Beijing: People's Daily Online.

[23] Gardner AS, Moholdt G, Cogley JG, Wouters B, Arendt AA, Wahr J, et al (2013) A reconciled estimate of glacier contributions to sea Level rise: 2003 to 2009. Science 340: 852–857. 10.1126/science.1234532 23687045

[24] Swenson S, Wahr J (2006) Post-processing removal of correlated errors in GRACE data. Geophysical Research Letters 33: L08402.

[25] Jiao JJ, Zhang X, Wang X (2015), Satellite-based estimates of groundwater depletion in the Badain Jaran Desert, China. Scientific Reports 5: 8960 10.1038/srep08960 25760683 PMC5390913

[26] Phan VH, Lindenbergh R, Menenti M (2012) ICESat derived elevation changes of Tibetan lakes between 2003 and 2009. International Journal of Applied Earth Observation and Geoinformation 17: 12–22.

[27] Yuan WH, Yin DW, Finlayson B, Chen ZY (2012) Assessing the potential for change in the middle Yangtze River channel following impoundment of the Three Gorges Dam. Geomorphology 147: 27–34.

[28] Voss KA, Famiglietti JS, Lo MH, de Linage C, Rodell M, Swenson SC (2013) Groundwater depletion in the Middle East from GRACE with implications for transboundary water management in the Tigris-Euphrates-Western Iran region. Water Resources Research 49: 904–914. 23658469 10.1002/wrcr.20078 PMC3644870

[29] Rodell M, Chen JL, Kato H, Famiglietti JS, Nigro J, Wilson CR (2007) Estimating groundwater storage changes in the Mississippi River basin (USA) using GRACE. Hydrogeology Journal 15: 159–166.

[30] Zhang L, Su F, Yang D, Hao Z, Tong K (2013) Discharge regime and simulation for the upstream of major rivers over Tibetan Plateau. Journal of Geophysical Research: Atmospheres, 118: 8500–8518.

[31] Schaner N, Voisin N, Nijssen B, Lettenmaier DP (2012) The contribution of glacier melt to streamflow. Environmental Research Letters 7: 034029.

[32] Pokhrel YN, Fan Y, Miguez-Macho G, Yeh PJF, Han SC (2013) The role of groundwater in the Amazon water cycle: 3. Influence on terrestrial water storage computations and comparison with GRACE. Journal of Geophysical Research-Atmospheres 118: 3233–3244.

[33] Zhang G, Xie H, Kang S, Yi D, Ackley SF (2011) Monitoring lake level changes on the Tibetan Plateau using ICESat altimetry data (2003–2009). Remote Sensing of Environment 115: 1733–1742.

[34] Qinghai Provincial Climate Monitoring and Assessment Centre (2009) Report on the Assessment of Climate Change in the Qaidam Basin. Qinghai Province.

[35] Wu TH, Li SX, Cheng GD, Nan ZT (2005) Using ground-penetrating radar to detect permafrost degradation in the northern limit of permafrost on the Tibetan Plateau. Cold Regions Science and Technology 41: 211–219.

[36] Song CQ, Huang B, Richards K, Ke LH, Phan VH (2014) Accelerated lake expansion on the Tibetan Plateau in the 2000s: Induced by glacial melting or other processes? Water Resources Research 50: 3170–3186.

[37] Gao Y, Li X, Leung LR, Chen D, Xu J (2015) Aridity changes in the Tibetan Plateau in a warming climate. Environmental Research Letters 10: 034013.

[38] Chen S, Liu Y, Thomas A (2006) Climatic change on the Tibetan Plateau: Potential evapotranspiration trends from 1961–2000. Climatic Change 76: 291–319.

[39] Gao G, Chen D, Ren G, Chen Y, Liao Y (2006) Spatial and temporal variations and controlling factors of potential evapotranspiration in China: 1956–2000. Journal of Geogrphical Sciences 16: 3–12.

[40] Zhang Y, Liu C, Tang Y, Yang Y (2007) Trends in pan evaporation and reference and actual evapotranspiration across the Tibetan Plateau. Journal of Geophysical Research 112: D12110.

[41] Cheng G, Wu T (2007) Responses of permafrost to climate change and their environmental significance, Qinghai-Tibet Plateau. Journal of Geophysical Research 112: F02S03.

[42] Ran Y, Li X, Cheng G, Zhang T, Wu Q, Jin H, et al (2012) Distribution of permafrost in China: an overview of existing permafrost maps. Permafrost and Periglacial Processes 23: 322–333.

[43] Cheng G, Jin H (2013) Permafrost and groundwater on the Qinghai-Tibet Plateau and in northeast China. Hydgrogeology Journal 21: 5–23.

[44] Jin H, He R, Cheng G, Wu Q, Wang S, Lü L, et al (2009) Changes in frozen ground in the Source Area of the Yellow River on the Qinghai–Tibet Plateau, China, and their eco-environmental impacts. Environmental Research Letters 4: 045206.

[45] Zhang X, He J, Zhang J, Polyakov I, Gerdes R, Inoue J, et al (2013), Enhanced poleward moisture transport and amplified northern high-latitude wetting trend. Nature Climate Change 3: 47–51.

[46] Williams JR (1970) Ground water in the permafrost regions of Alaska. U. S. Geological Survey Professional Paper 696.

[47] Fasullo JT, Boening C, Landerer FW, Nerem RS (2013) Australia’s unique influence on global sea level in 2010–2011. Geophysical Research Letters 40: 4368–4373.

